# Association of urinary albumin-to-creatinine ratio with cardiometabolic risk markers and pre-diabetes in adults with normoglycemia, normoalbuminuria, and normotension with parental type 2 diabetes

**DOI:** 10.1136/bmjdrc-2023-003609

**Published:** 2024-01-17

**Authors:** Matthew Everett, Natasha Rushing, Peace Asuzu, Jim Wan, Samuel Dagogo-Jack

**Affiliations:** 1 Division of Endocrinology, Diabetes and Metabolism, University of Tennessee Health Science Center College of Medicine, Memphis, Tennessee, USA; 2 Preventive Medicine, University of Tennessee Health Science Center College of Medicine, Memphis, Tennessee, USA; 3 General Clinical Research Center, University of Tennessee Health Science Center College of Medicine, Memphis, Tennessee, USA

**Keywords:** Albuminuria, Impaired Fasting Glucose, Impaired Glucose Tolerance, Ethnic Groups

## Abstract

**Introduction:**

This is a post hoc analysis of urinary albumin-to-creatinine ratio (uACR) within the normoalbuminuric range in relation to cardiometabolic risk factors among initially normoglycemic, normotensive participants in the Pathobiology of Prediabetes in a Biracial Cohort (POP-ABC) Study.

**Research design and methods:**

308 healthy African American (AA) and European American (EA) participants in the POP-ABC Study underwent baseline assessments, including oral glucose tolerance test, anthropometry, urinary albumin-to-creatinine ratio (uACR), lipids, adipocytokines, insulin sensitivity and secretion. Participants were followed quarterly for 5.5 years (mean 2.62 years) for the primary outcome of incident pre-diabetes.

**Results:**

The cohort’s mean fasting glucose was 92.1±6.90 mg/dL, 2-hour plasma glucose was 123±25.0 mg/dL, systolic blood pressure was 123±15.9 mm Hg, and diastolic blood pressure was 74±8.80 mm Hg. Baseline uACR levels (range 1–29 mg/g) were similar in AA versus EA participants (6.40 mg/g±4.80 vs 6.80±5.40 mg/g, p=0.52), higher in women than men (7.30 mg/g±5.30 vs 4.60±3.90 mg/g, p<0.0001), and showed significant associations with cardiometabolic risk factors, including age, insulin sensitivity, high-density lipoprotein cholesterol, and adiponectin levels (p=0.03–0.004). During 5.5 years of follow-up, 104 participants developed pre-diabetes and 204 maintained normoglycemia. Baseline uACR quartiles were associated with incident pre-diabetes (r=0.19, p=0.0011).

**Conclusions:**

Baseline uACR levels were associated with cardiometabolic risk markers and incident pre-diabetes risk among adults with normoglycemia, normoalbuminuria and normotension with parental diabetes.

**WHAT IS ALREADY KNOWN ON THIS TOPIC**

- Previous studies have established higher-grade albuminuria as a marker of cardiorenal complications in people with diabetes.
- However, few studies have examined the significance of urinary albumin excretion within the normoalbuminuric range among otherwise healthy individuals.

**WHAT THIS STUDY ADDS**

- We studied healthy, normoglycemic, normotensive African Americans and European Americans with normal urinary albumin-to-creatinine ratio (uACR) (≤29 mg/g creatinine).
- uACR levels within the normal range showed significant associations with cardiometabolic variables, including age, insulin sensitivity, high-density lipoprotein cholesterol, adiponectin, and incident pre-diabetes.

**HOW THIS STUDY MIGHT AFFECT RESEARCH, PRACTICE OR POLICY**

- These findings expand current understanding by demonstrating associations between cardiometabolic markers and levels of albuminuria that are well within the normal range.
- The observed association between low-grade albuminuria and adiponectin levels suggests an inflammatory mechanism.
- Further studies are needed to elucidate the mechanisms and pathophysiological significance of the link between cardiometabolic risk factors and low-grade albuminuria.

## Introduction

Increased urinary excretion of albumin is a marker of endothelial dysfunction and cardiometabolic and renal risk, especially in people with diabetes or hypertension.^1–4^ Even modest changes in urine albumin-to-creatinine ratio (uACR) within the normal range have been associated with increased risks of hypertension and cardiovascular risk.^5 6^ Elevated urinary albumin excretion has been associated with increased risk of new-onset diabetes, but it is unknown whether the link between albuminuria and glucose dysregulation precedes diabetes diagnosis.^2^ Pre-diabetes, defined as impaired fasting glucose (IFG) or impaired glucose tolerance (IGT), represents an intermediate stage in the progression from normal glucose regulation to type 2 diabetes.^7^ The risk factors for pre-diabetes overlap considerably with those of type 2 diabetes.^8^ Given the reported association between albuminuria with endothelial dysfunction, cardiometabolic risk, and new-onset diabetes,^2 9–11^ we determined whether a more proximal relationship exists between albuminuria and early dysglycemia.

Because urinary albumin excretion is a continuous variable, with a known conventional upper limit, we were interested in determining whether albuminuria within the normal reference range is associated with cardiometabolic risk markers. Specifically, we tested the hypothesis that albumin excretion within the normal reference range among normoglycemic, normotensive persons is associated with cardiometabolic risk markers and pre-diabetes. We tested our hypothesis in a large population of initially normoglycemic black or African American and white or European American participants enrolled in the Pathobiology of Prediabetes in a Biracial Cohort (POP-ABC) Study.^12–14^ The POP-ABC participants were followed up at quarterly intervals for 5.5 years, the primary outcome being progression from normoglycemia to incident pre-diabetes.^12–14^ Using this prospective cohort, we have documented the association between urinary albumin excretion at enrollment and some cardiometabolic risk markers and incident pre-diabetes among initially normoglycemic, normotensive and normoalbuminuric study participants.

## Research design and methods

### Study subjects

The POP-ABC Study enrolled individuals with self-reported non-Hispanic African American (black) or European American (white) status, aged 18–65 years, who have one or both biological parents with type 2 diabetes. In addition to parental diabetes, eligible subjects were required to have normal fasting plasma glucose (FPG) (<100 mg/dL (5.6 mmol/L)) and/or normal 2-hour plasma glucose (2hrPG) (<140 mg/dL (7.8 mmol/L)) during oral glucose tolerance test (OGTT), as previously described.^12–14^ Excluded from participation were those with a diagnosis of diabetes, acute illness or uncontrolled hypertension, and individuals taking medications known to alter blood glucose, insulin secretion, insulin sensitivity, or body weight. The main report from the POP-ABC Study showed no racial differences in incident pre-diabetes or glycemic progression among 343 participants with parental history of diabetes during 5.5 years of follow-up.^14^ The present report is a post hoc analysis of the association of baseline uACR levels within the normoalbuminuric range in relation to cardiometabolic risk factors among initially normoglycemic and normotensive POP-ABC participants. Participants (N=308) with baseline uACR levels of 29 mg/g or lower, who had normal blood pressure (120/80 mm Hg or lower), normoglycemia, and evaluable follow-up data were included in the present analysis. A baseline uACR >30 mg/g and use of medications known to alter albumin excretion were additional exclusion criteria for this post hoc study.

### Clinical evaluation

The University of Tennessee General Clinical Research Center (GCRC) was the study performance site. Participants arrived after an overnight fast and underwent baseline assessments, including medical interview, clinical examination, spot urine specimen albumin and creatinine measurement, blood chemistry, and a standard 75-gram OGTT.^12–14^ Height, weight and waist circumference were measured, and the body mass index (BMI) was calculated as the weight (kg) divided by height in meter squared. After the baseline visit, enrolled participants made follow-up visits to the GCRC for prespecified assessments, including anthropometry, blood pressure, FPG and biochemical measurements every 3 months. Participants also underwent OGTT annually (or at occurrence of endpoint), as previously described.^12–14^

We measured whole-body insulin sensitivity with the hyperinsulinemic-euglycemic clamp and insulin secretion with the frequently sampled intravenous glucose tolerance test, as previously described.^12–14^ The insulin sensitivity index was derived from the steady-state glucose disposal rate corrected for the steady-state plasma insulin levels.^12–15^ The acute insulin response was calculated as the mean incremental plasma insulin level at 3, 4 and 5 min after administration of an intravenous bolus of dextrose (25 g).^12–14^

### Biochemical measurements

Plasma glucose was measured using a glucose oxidase method (Yellow Springs Instruments Co, Yellow Springs, Ohio, USA). Hemoglobin A1c (HbA1c), fasting plasma lipid profiles and urinary albumin and creatinine levels were measured in a contract clinical laboratory using standard methodologies. Plasma insulin was measured in our Endocrine Research Laboratory using a chemiluminescent assay, using commercial kits (Immulite, Siemens, Llanberis, Gwynedd, UK). The sensitivity of the insulin assay was 2 μIU/mL and the within-run and between-run coefficients of variation were 4.7% and 8%, respectively.

### Definition of outcome

Participants were followed quarterly at the GCRC for 5.5 years (mean 2.62 years). The primary outcome was the occurrence of pre-diabetes, as indicated by IFG (FPG 100–125 mg/dL) or IGT (2hrPG 140–199 mg/dL) based on American Diabetes Association criteria.^6^ Confirmatory of endpoint was performed using a standard 75-gram OGTT, usually performed within 6 weeks of initial endpoint occurrence. All endpoints were adjudicated independently by the Institutional Data and Safety Officer (Murray Heimberg).

### Statistical analysis

We report data as means±SD. Continuous variables were analyzed using unpaired t-test, and categorical variables by Χ^2^ test. The distributions of uACR by sex and ethnicity were analyzed using the Kolmogorov-Smirnov test. Associations between defined groups were analyzed using Wilcoxon rank-sum test and Pearson’s or Spearman’s correlation, as appropriate. Logistic regression models were used to analyze uACR as a predictor of incident pre-diabetes, after adjustments for age, sex, race/ethnicity, BMI, and plasma glucose. All statistical analyses were performed with the use of Statview V.5.0 statistical software (SAS Institute, Cary, North Carolina, USA).

## Results

For the present report, we included all 308 offspring of parents with type 2 diabetes mellitus (167 African Americans, 141 European Americans) enrolled in the POP-ABC Study with baseline uACR values <30 mg/g who had evaluable follow-up data. Table 1 shows the baseline characteristics of the study participants.

**Table 1:** Comparison of baseline characteristics of study participants by ethnicity

	All participants (female/male)	African American (female/male)	European American (female/male)	P value
Number	308 (221/87)	167 (125/42)	141 (96/45)	
Age (years)	45.1±10.3	43.3±10.0	47.2±10.3	0.0009
Waist circumference (cm)	94.7±15.8	96.3±16.1	92.7±15.3	0.04
BMI (kg/m^2^)	30.3±7.30	31.7±7.70	28.6±6.40	0.0002
SBP (mm Hg)	123±15.9	125±17.0	121±14.1	0.04
DBP (mm Hg)	74±8.80	74±9.10	73±8.40	0.10
Triglycerides (mg/dL)	95.2±55.7	78.1±38.3	115.7±65.6	<0.0001
HDL cholesterol (mg/dL)	52.2±14.0	52.8±14.6	51.5±13.1	0.43
Urinary albumin-to-creatinine ratio (mg/g)	6.60±5.10	6.40±4.80	6.80±5.40	0.52
FPG (mg/dL)	92.1±6.90	90.9±7.10	93.6±6.40	0.0005
hsCRP (mg/L)	3.90±6.00	4.80±6.80	2.8±4.60	0.003
Adiponectin (μg/mL)	9.44±5.35	8.39±5.00	10.7±5.50	0.0002
HbA1c (%)	5.55±0.45	5.65±0.48	5.44±0.39	<0.0001
Acute insulin response (uU/mL)	84.3±72.5	106±87.4	60.6±39.4	<0.0001
Insulin sensitivity (μmol/kg/min/pM)	0.133±0.067	0.123±0.069	0.144±0.063	0.02

To convert values for glucose to mmol/L, multiply by 0.0555. To convert values for triglycerides to mmol/L, multiply by 0.01129. To convert values for cholesterol to mmol/L, multiply by 0.02586. To convert the values for insulin to pmol/L, multiply by 6.0.

BMI, body mass index; DBP, diastolic blood pressure; FPG, fasting plasma glucose; HbA1c, hemoglobin A1c; HDL, high-density lipoprotein; hsCRP, high-sensitivity C reactive protein; SBP, systolic blood pressure.

The participants’ mean age was 45.1±10.3 years, BMI was 30.3±7.28 kg/m^2^, systolic blood pressure was 123±15.9 mm Hg, and diastolic blood pressure was 74±8.80 mm Hg. The FPG was 92.1±6.89 mg/dL, 2hrPG was 123±25.0 mg/dL, and HbA1c was 5.55±0.45%. The mean values for blood pressure, BMI, C reactive protein, waist circumference, acute insulin response, and HbA1c were higher, and those for FPG, triglycerides, adiponectin, and insulin sensitivity were lower, in African American compared with European American participants (table 1).

### Distribution of uACR

**Figure 1:**
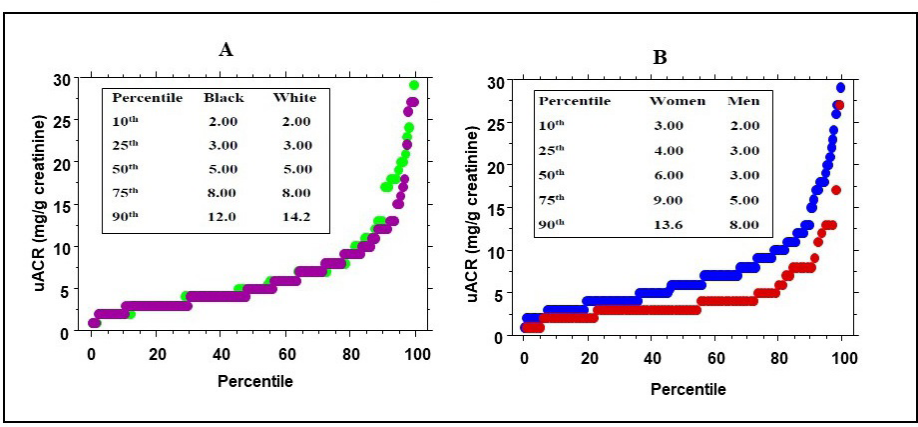
Percentile distribution of urinary albumin-to-creatinine ratio (uACR) in (A) black (purple symbol) and white (green symbol) participants and (B) female (blue symbol) and male (red symbol) participants in the POP-ABC Study. uACR distribution was similar in black versus white participants but significantly higher in women than men (p<0.0001). POP-ABC, Pathobiology of Prediabetes in a Biracial Cohort.

By design, we excluded those POP-ABC Study participants (N=35) whose uACR was >30 mg/g at baseline. Thus, the baseline uACR in the present study ranged from 1 to 29 mg/g creatinine; the mean uACR was 6.55 mg/g and the median value was 5 mg/g. The mean uACR was higher in women than men (7.30 mg/g±5.30 vs 4.60 ± 3.90 mg/g, p<0.0001) but similar in African American versus European American participants (6.4 mg/g±4.8 vs 6.8±5.4 mg/g, p=0.52). Figure 1 shows the percentile distribution of uACR for all subjects and by sex and ethnicity. The distribution of uACR values was similar in African American and European American participants but differed significantly by sex, with higher values in women than men (p<0.001) (figure 1).

### Cardiometabolic correlates of uACR

**Figure 2:**
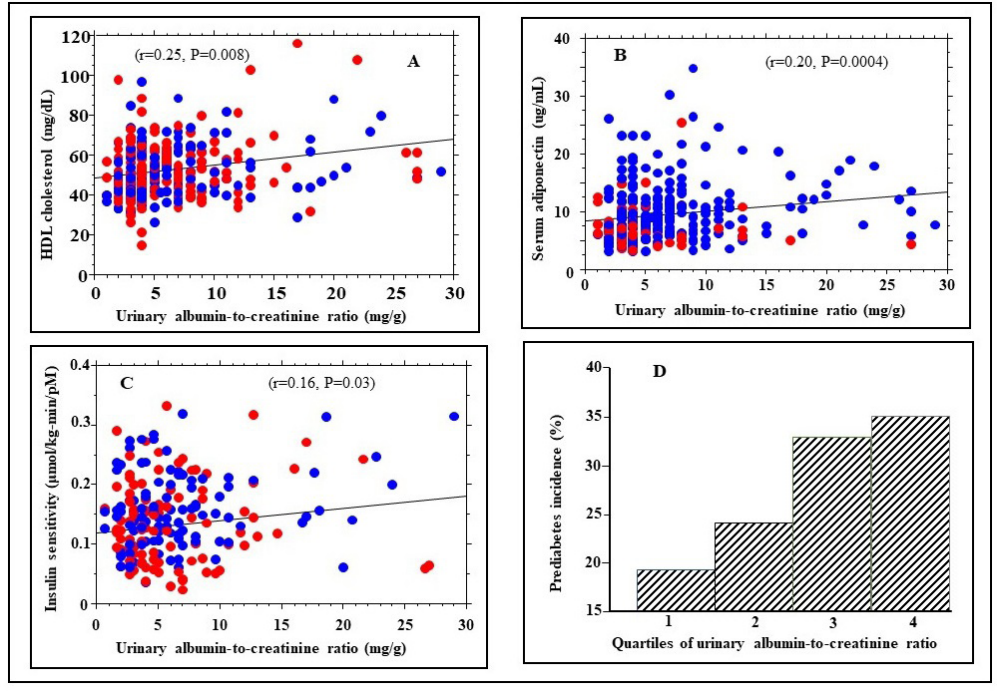
Association of urinary albumin-to-creatinine ratio (uACR) with (A) serum high-density lipoprotein (HDL) cholesterol levels, (B) serum adiponectin levels and (C) insulin sensitivity in African American (red symbols) and European American (blue symbols) study participants; (D) pre-diabetes incidence across quartiles of baseline uACR among female study participants (uACR quartiles (mg/g): Q1: 0–<4; Q2: 4–<6; Q3: 6–<9; Q4: 9–<30).

We observed univariate associations between uACR and some cardiometabolic risk markers, including age (r=0.16, p=0.007). Table 2 and figure 2 show associations of uACR levels with adiposity measures, blood pressure, lipid profile, plasma glucose, inflammatory markers, and insulin sensitivity. Baseline uACR levels correlated with plasma levels of high-density lipoprotein (HDL) cholesterol (r=0.25, p=0.0004), adiponectin (r=0.20, p=0.0004), and whole-body insulin sensitivity (r=0.16, p=0.03) (figure 2A–C).

**Table 2:** Association of urinary albumin-to-creatinine ratio with cardiometabolic risk markers*

	All participants (N=308)		African American (N=16)		European American (N=141)	
	r	P value	r	P value	r	P value
BMI (kg/m^2^)	0.04	0.44	0.005	0.95	0.08	0.34
Waist circumference (cm)	0.1	0.07	0.005	0.94	−0.22	0.009
SBP (mm Hg)	0.005	0.93	0.04	0.63	0.05	0.56
DBP (mm Hg)	0.06	0.27	0.02	0.83	0.15	0.07
Triglycerides (mg/dL)	0.06	0.27	0.12	0.11	0.01	0.89
HDL-c (mg/dL)	0.25	0.008	0.21	0.005	0.22	0.01
LDL-c (mg/dL)	0.03	0.55	0.002	0.98	0.15	0.07
HbA1c (%)	0.09	0.13	0.07	0.37	0.1	0.26
FPG (mg/dL)	0.02	0.75	0.05	0.5	0.12	0.16
2hrPG (mg/dL)	0.07	0.25	0.008	0.92	0.14	0.09
Adiponectin (μg/mL)	0.2	0.0004	0.21	0.008	0.19	0.03
hsCRP (mg/L)	0.003	0.96	0.04	0.6	0.05	0.59
Insulin sensitivity (μmol/kg/min/pM)	0.16	0.03	0.06	0.53	0.23	0.02

To convert values for glucose to mmol/L, multiply by 0.0555. To convert values for triglycerides to mmol/L, multiply by 0.01129. To convert values for cholesterol to mmol/L, multiply by 0.02586. To convert values for insulin to pmol/L, multiply by 6.0.

*All variables were assessed at baseline following enrollment in the POP-ABC Study.

BMI, body mass index; DBP, diastolic blood pressure; FPG, fasting plasma glucose; HbA1c, hemoglobin A1c; HDL-c, high-density lipoprotein cholesterol; 2hrPG, 2-hour plasma glucose after 75 g oral glucose challenge; hsCRP, high-sensitivity C reactive protein; LDL-c, low-density lipoprotein cholesterol; POP-ABC, Pathobiology of Prediabetes in a Biracial Cohort; SBP, systolic blood pressure.

Notably, the association of uACR with waist circumference and insulin sensitivity was evident in European American but not African American participants (table 2). In contrast, the association of uACR with HDL cholesterol and adiponectin levels was observed in African American (r=0.21, p=0.005) and European American (r=0.22, p=0.01) participants (table 2). As serum HDL cholesterol and adiponectin levels differ significantly in men and women, we ran sex-specific analyses to confirm their association with uACR. The association of uACR with HDL cholesterol was evident in women (r=0.14, p=0.04) and men (r=0.20, p=0.05), whereas that between uACR and serum adiponectin was observed in women (r=0.17, p=0.009) but not men (r=0.018, p=0.87). Serum triglycerides showed no correlation with uACR in the overall study population; however, a significant correlation was observed in women (r=0.16, p=0.02) but not men (r=0.02, p=0.85). No significant associations were observed between baseline uACR and other cardiometabolic risk markers, including blood pressure, low-density lipoprotein cholesterol, plasma glucose, or C reactive protein (CRP) levels (table 2).

### Baseline uACR quartiles and progression to pre-diabetes

During 5.5 years of follow-up (mean 2.62 years) in the POP-ABC Study, 104 participants (34%) developed pre-diabetes (progressors), and 204 participants (66%) maintained normoglycemia (non-progressors). The baseline clinical characteristics of individuals who progressed to pre-diabetes versus non-progressors have been published previously in the main report from the POP-ABC Study.^14^ In brief, progressors were older (47.6±9.0 years vs 43.8±10.7 years, p=0.0018) and had higher FPG (94.0±7.20 mg/dL vs 91.1±6.6 mg/dL, 0.0005) and 2hrPG (128±25 mg/dL vs 121±20 mg/dL, p=0.011) values at baseline compared with non-progressors. However, baseline BMI was not significantly different in progressors versus non-progressors (31.2±6.6 kg/m^2^ vs 29.7±7.5 kg/m^2^, p=0.085).

We stratified participants by quartiles of baseline uACR as follows: quartile 1: 0–<4 mg/g (N=90; 42 women, 48 men); quartile 2: 4–<6 mg/g (N=80; 58 women, 22 men); quartile 3: 6–<9 mg/g (N=70; 61 women, 9 men); and quartile 4: 9–<30 mg/g (N=68; 60 women, 8 men). We observed a significant association between uACR quartiles at baseline and the occurrence of pre-diabetes (ρ=0.19, p=0.001). A Wilcoxon signed-rank test showed a significant difference (Z=−11.234, p<0.0001) in baseline uACR quartiles between progressors to pre-diabetes and non-progressors. Higher baseline uACR quartiles were associated with higher risk of progression to pre-diabetes in women (Z=−10.965, p<0.0001) and men (Z=−2.191, p=0.02). Figure 2D shows incident pre-diabetes rates across quartiles 1–4 of uACR among female participants. A similar pattern was seen among men from quartile 1 to 2, but samples were too few in the upper quartile (nine men and eight men in quartiles 3 and 4, respectively).

In unadjusted, minimally adjusted, and fully adjusted logistic regression models, the odds of incident pre-diabetes increased numerically across increasing quartiles of baseline uACR, with wide confidence (table 3).

**Table 3:** Logistic regression of baseline uACR quartiles and incident pre-diabetes

**Unadjusted**		
**uACR quartiles (referent=quartile 1)**	**OR**	**95% CI**
Quartile 2	1.067	0.427 to 2.668
Quartile 3	1.138	0.432 to 2.998
Quartile 4	2.582	1.044 to 6.283
**Model 1**		
**uACR quartiles (referent=quartile 1)**	**OR**	**95% CI**
Quartile 2	1.351	0.675 to 2.704
Quartile 3	1.544	0.732 to 3.254
Quartile 4	1.765	0.834 to 3.736
**Model 2**		
**uACR quartiles (referent=quartile 1)**	**OR**	**95% CI**
Quartile 2	1.366	0.661 to 2.821
Quartile 3	1.613	0.733 to 3.553
Quartile 4	1.929	0.876 to 4.25

Model 1: adjusted for age, ethnicity, and sex; model 2: adjusted for model 1 and BMI, waist circumference, and blood glucose.

BMI, body mass index; uACR, urinary albumin-to-creatinine ratio.

## Discussion

Studies in the literature focus predominantly on higher-grade albuminuria as evidence of diabetic nephropathy, a complication of hypertension, or a marker of cardiorenal disease.^1–4 8–10 15–23^ Elevated albuminuria has also been associated with risks of endothelial dysfunction, cardiovascular death, and all-cause mortality.^1 5 6^ The novelty of our present study was the extension of knowledge to less severe degrees of albuminuria. We observed significant (although modest) associations between uACR levels within the normal range and several cardiometabolic risk markers (age, insulin sensitivity, HDL cholesterol and adiponectin) among otherwise healthy normoglycemic, normotensive individuals.

Furthermore, we explored the association between baseline uACR and the risk of progression from normoglycemia to pre-diabetes. Given the numerous behavioral and biochemical risk factors for glucose dysregulation, we were surprised to find significant correlations between baseline uACR levels and incident pre-diabetes, using measures of either monotonic relationship (Spearman rank correlation) or stochastic dominance (Wilcoxon rank-sum test). Further testing in logistic regression models showed consistently higher odds of pre-diabetes with higher uACR quartiles, although with wide 95% CIs (particularly in the adjusted models) probably due to modest sample size and variability in uACR values. Our findings suggest that uACR levels within the normal range may not be entirely benign. Indeed, previous studies have reported associations between uACR levels within the normal range (<30 mg/g) and hypertension and cardiorenal disease in the general population.^5 6^

Our study cohort comprising male and female African American and European American participants allowed us to examine clinical and cardiometabolic correlates of uACR across sex and ethnicity. We observed significant differences in uACR by sex but not ethnicity: values were higher in women than men. Our finding of higher uACR levels in women than men is consistent with previous reports.^24 25^ The lower uACR values in men are likely explained by the higher muscle mass (and consequently higher creatinine excretion) in men relative to women.^25^ In contrast to the lack of ethnic disparities in uACR levels in the present study, previous studies had reported higher levels of urinary albumin excretion in African American individuals than European American individuals.^25 26^ The discrepancies might be related to differences in methodology as well as phenotypical characteristics and genetic background of the populations studied. Unlike the previous studies, our present report is based on a cohort of healthy African Americans and European Americans with similar parental history of type 2 diabetes, all of whom had uACR <30 mg/dL, normal blood pressure, and normal blood glucose levels at enrollment.

Baseline uACR levels correlated significantly with age and cardiometabolic risk markers, such as waist circumference, HDL cholesterol, adiponectin, and insulin sensitivity. A population-based study in China reported a selective association of uACR with serum triglycerides but not other lipid moieties.^27^ In the present study, the association of uACR with serum triglycerides was observed only among women. Our observation of a direct correlation between uACR and HDL cholesterol levels in the normoglycemic POP-ABC population is discordant with a previous report of an inverse association in people with type 1 diabetes.^28^ Insulin deficiency decreases HDL cholesterol levels, which might explain differences in findings from a healthy cohort versus patients with type 1 diabetes.^29^

Besides the association with HDL cholesterol levels, we found that baseline uACR levels were positively correlated with insulin sensitivity and serum adiponectin levels. Adiponectin levels have been reported to correlate either directly^30 31^ or inversely^32 33^ with albuminuria. A direct association between uACR levels and adiponectin was reported in studies that enrolled patients with chronic kidney disease^30^ or diabetic nephropathy,^31^ and an inverse correlation was reported in studies that enrolled individuals with overweight or obesity but without diabetes or known kidney disease.^32 33^

Higher uACR levels portend unfavorable traits, including endothelial dysfunction, inflammation, cardiometabolic and renal risks,^1–5 8–10 15–23^ whereas higher levels of insulin sensitivity, HDL cholesterol, and adiponectin are beneficial cardiometabolic attributes. Thus, our finding of direct associations between uACR levels and insulin sensitivity, HDL cholesterol, and adiponectin would seem paradoxical. Although the exact mechanisms are unclear, we would speculate that our overall findings might be congruent if an upward drift in uACR within the normal range triggered compensatory mechanisms that involve a beneficial augmentation of adiponectin, HDL cholesterol, and insulin sensitivity. The lack of association between CRP and uACR in our study cohort probably reflects the normoalbuminuric, normoglycemic and normotensive status of the study participants. Association of albuminuria with CRP tends to be on a background of established diabetes, hypertension, or chronic kidney disease.^34 35^ The unique feature of our study is that we found meaningful associations between certain cardiometabolic risk markers and uACR values within the normal reference range in people without hyperglycemia, hypertension, or evidence of kidney disease.

Adiponectin is a vasoactive, anti-inflammatory, anti-oxidant, insulin-sensitizing and cardioprotective adipocytokine that exerts its effect, at least in part, by activating the AMP-activated protein kinase pathway.^36 37^ Adiponectin-knockout mice exhibit impairments in kidney structure and function, including fusion of glomerular podocytes and increased urinary albumin excretion.^32^ These defects are reversed by treatment with adiponectin.^33^ Thus, it is conceivable that a compensatory increase in adipocyte synthesis and secretion of adiponectin could be triggered in the setting of subclinical inflammation, nascent endothelial dysfunction or other putative factors that increase albuminuria.^38^ The augmented adiponectin output, ostensibly, is aimed at restoring endothelial, cardiorenal, and metabolic homeostasis via stimulation of insulin sensitivity and inhibition of toxic inflammatory and oxidative pathways.

In our study, individuals at the upper quartiles of baseline uACR levels were more likely to progress from normoglycemia to pre-diabetes compared with those at the lowest quartile. Plausible pathophysiological mechanisms linking higher uACR to dysglycemia include inflammation, oxidative stress, endothelial dysfunction, lipotoxicity, hypertension, and insulin resistance.^38–42^ Higher-grade albuminuria (30–300 µg/g) indicates incipient or established kidney disease and gross proteinuria (>300 mg/g) signifies advanced disease and increased risks of adverse clinical events. Our present findings expand current understanding by demonstrating that even low-grade albuminuria within the normal range may be associated with pre-diabetes risk in a diverse prospective cohort. Importantly, both low-grade albuminuria and pre-diabetes are known to be associated with increased risks of vascular complications.^8 9^

The strengths of our study include the enrollment of a diverse cohort of African American and European American participants and the longitudinal design. Other strengths include the rigorous ascertainment of pre-diabetes events; the restriction of analysis to individuals with normal uACR values; and the exclusion of individuals with higher-grade albuminuria, hypertension, and medical conditions likely to confound the interpretation of results.

One limitation of the present study is the requirement that all POP-ABC Study participants have one or both biological parents with type 2 diabetes. The restriction of enrollment to offspring of parents with type 2 diabetes does limit the generalizability of our findings. Another limitation is the small sample size of men in the upper two quartiles of uACR, which reflected sex differences in the distribution of uACR. The total male participants in the upper two uACR quartiles of 17 compared with 121 female participants in those same quartiles meant that we had to rely on female participants to discern the full spectrum of incident diabetes risk across uACR quartiles. Furthermore, the use of a single (random) urine specimen for uACR measurement, though standard clinical practice, is a weakness of the present study. Furthermore, the observed correlation of uACR with several cardiometabolic measures (such as waist circumference and insulin sensitivity) was modest, which indicates that additional factors contribute to the variance in the values for these biological measures. Similarly, the wide 95% CIs around the point estimates for the odds of pre-diabetes indicate that the association of uACR levels with pre-diabetes risk is not independent of other factors.

In conclusion, in our high-risk cohort of African American and European American offspring of parents with type 2 diabetes, albumin excretion within the normal range at enrollment showed associations with cardiometabolic risk markers and the risk of progression from normoglycemia to pre-diabetes.

## Data Availability

Data are available upon reasonable request.
